# Case Report: ALK-positive histiocytosis with a novel *PTRH2::ALK* fusion masquerading as a liver abscess in an infant

**DOI:** 10.3389/fimmu.2026.1722061

**Published:** 2026-04-29

**Authors:** Qian Wan, Hui Huang, Zhongjin Xu, Caihui Yuan, Yangyang Ma, Chongjun Wu

**Affiliations:** 1 Department of Hematology, Jiangxi Provincial Children’s Hospital, Nanchang, Jiangxi, China; 2 Department of Pathology, Jiangxi Provincial Children’s Hospital, Nanchang, Jiangxi, China; 3 Nuclear Magnetic Resonance Room, Jiangxi Provincial Children’s Hospital, Nanchang, Jiangxi, China; 4 Department of Pathology, Children’s Hospital of Fudan University, National Children’s Medical Center, Shanghai, China

**Keywords:** ALK-positive, histiocytosis, infant, liver abscess, PTRH2::ALK fusion

## Abstract

**Background:**

ALK-positive histiocytosis is a rare form of cutaneous histiocytosis and has been explicitly designated as a novel histiocytic neoplasm in recent classification systems. This disease may involve multiple organs throughout the body. Its diagnosis primarily relies on pathological confirmation, and molecular evidence of *ALK* rearrangement is required to establish a definitive diagnosis. For patients with unresectable or disseminated ALK-positive histiocytosis, therapy with ALK inhibitors has demonstrated considerable efficacy.

**Case presentation:**

An 8-month-old Chinese male infant was admitted with 1-day history of fever and newly diagnosed anemia of 0.5-day duration. Physical examination revealed chronic rashes on the bilateral lower extremities, including the plantar surfaces. Laboratory investigations demonstrated anemia (hemoglobin level: 63 g/L), elevated inflammatory markers, and an increased serum S-100 protein level (9.11 ug/L). Abdominal ultrasonography and contrast-enhanced computed tomography (CT) revealed hepatosplenomegaly and diffuse hypoechoic or cystic low-density lesions in the liver (largest approximately 7 × 8 mm), which were initially suspected to represent a liver abscess. Histopathological examination of a biopsy specimen from the lower extremity rash revealed dermal proliferation of histiocytes, foamy cells, and short spindle cells, accompanied by scattered multinucleated giant cells and lymphocytes. Immunohistochemical analysis demonstrated positivity for CD68, S100, and ALK, and negativity for CD1a, CD3, CD20, and other markers. Fluorescence *in situ* hybridization (FISH) confirmed *ALK* rearrangement, and next-generation sequencing (NGS) identified a novel *PTRH2::ALK* fusion. Positron emission tomography–computed tomography (PET-CT) subsequently revealed mediastinal space-occupying lesions, multiple hypermetabolic lesions in the liver, kidney, mesentery, and bones, as well as metabolically active systemic lymph nodes, findings consistent with ALK-positive histiocytosis. The patient remained clinically stable during follow-up after initiation of the ALK inhibitor crizotinib.

**Conclusion:**

This study reports a rare case of infantile ALK-positive histiocytosis that was initially misdiagnosed as a liver abscess and identifies a novel *PTRH2::ALK* fusion gene. In addition, this study systematically summarizes, for the first time, the age, sex, lesion distribution, fusion genes, treatment strategies, and prognosis of all previously reported pediatric cases of ALK-positive histiocytosis.

## 1 Introduction

ALK-positive histiocytosis is a rare form of cutaneous histiocytosis that predominantly affects the pediatric population (1). It has been explicitly designated as a novel histiocytic neoplasm entity in the 5th edition of the World Health Organization (WHO) Classification of Haematolymphoid Tumours and the International Consensus Classification (ICC) (2). This disease was first described by Chan et al., who reported three infant patients presenting with pallor, massive hepatosplenomegaly, anemia, and thrombocytopenia (3). As of 2008, no more than 100 cases of ALK-positive histiocytosis had been reported in peer-reviewed clinical literature, including individual case reports (4, 5).

A defining feature of ALK-positive histiocytosis is the proliferation of foamy histiocytes-cells exhibiting a bland cytological phenotype, expressing the ALK protein, and harboring *ALK* gene rearrangements (3). Molecular characterization has identified recurrent *ALK* rearrangements, with *KIF5B::ALK* being the most frequently reported fusion transcript. Rare fusion partner genes include *COL1A2, TRIM33, TPM3, EML4, DCKN1*, and *VRK2 (*6).

Given the rarity and clinical heterogeneity of ALK-positive histiocytosis, we report the case of an 8-month-old male infant who was initially misdiagnosed as having a liver abscess. This case expands the molecular and diagnostic spectrum of ALK-positive histiocytosis by identifying a novel *PTRH2::ALK* fusion gene, which was confirmed through pathological examination of a skin punch biopsy specimen. In addition, this case highlights the importance of considering this rare histiocytic neoplasm in pediatric patients presenting with the triad of atypical persistent fever, maculopapular rash, and a non-tender hepatic mass, thereby reducing the risk of misdiagnosis as a treatable infectious disease, such as bacterial liver abscess.

## 2 Case presentation

**Figure 1:**
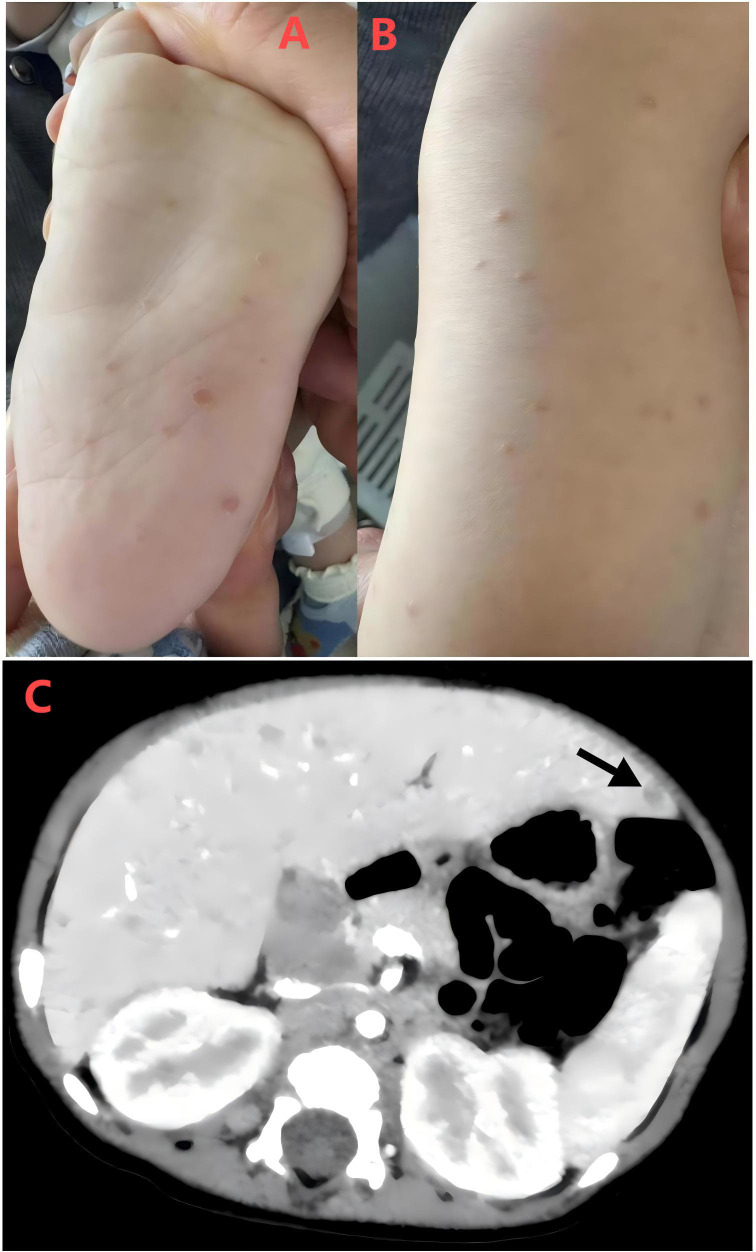
**(A)** Multiple reddish, round-to-oval macules on the infant’s sole, with some lesions exhibiting epidermal disruption on the surface. **(B)** Multiple well-circumscribed, erythematous, discrete papules distributed over the infant’s lower limbs. **(C)** Contrast-enhanced abdominal CT demonstrates diffuse cystic low-attenuation lesions within the liver (arrows). The largest lesion (~ 6 × 5 cm) shows ill-defined margins with mild peripheral enhancement, findings suggestive of an infectious process.

An 8-month-old male infant was admitted to our clinic with a 1-day history of fever and newly diagnosed anemia of 0.5-day duration. Physical examination revealed chronic rashes on the bilateral lower extremities, including the plantar surfaces (**Figures 1A, B**).

Laboratory investigations demonstrated leukocytosis, a normal platelet count, and iron-deficiency anemia, characterized by a hemoglobin level of 63 g/L, a mean corpuscular volume (MCV) of 67.9 fL, a mean corpuscular hemoglobin (MCH) of 19.5 pg, a reticulocyte count of 4.66%, a ferritin concentration of 1320.9 ng/mL (normal range: 14–142 ng/mL ), and a serum iron level of 3.2 μmol/L (normal range: 13-32 μmol/L). Serum folic acid and vitamin B12 levels, as well as thalassemia-related genetic testing, were within normal limits. Bone marrow cytology demonstrated proliferative anemia with erythroid hyperplasia, 1% intracellular iron, and grade + extracellular iron deposition.

Additional laboratory findings included normal renal, hepatic, and coagulation function; hypoalbuminemia (24.5 g/L); an elevated C-reactive protein level (CRP, 51.46 mg/L); an increased erythrocyte sedimentation rate (ESR, 40 mm/h); elevated interleukin-6 (IL-6) levels (43.76 pg/mL); and an increased interleukin-2 (IL-2) receptor level (6228 pg/mL). Serological tests for hepatitis B virus, syphilis, Epstein–Barr virus (EBV), and human immunodeficiency virus (HIV) were negative. Tumor markers, including alpha-fetoprotein (AFP) and carcinoembryonic antigen (CEA), were within normal ranges.

Abdominal ultrasonography demonstrated hepatomegaly (4.3 cm below the costal margin) and splenomegaly (2.3 cm below the costal margin). Diffuse discrete hypoechoic lesions were observed within the hepatic parenchyma, with the largest lesion measuring approximately 7 × 8 mm, raising suspicion for a liver abscess. Contrast-enhanced abdominal CT revealed diffuse cystic low-density lesions in the liver, with the largest lesion measuring approximately 6 × 5 mm and exhibiting ill-defined margins. Mild peripheral enhancement was observed after contrast administration, further supporting an initial diagnosis of an infectious etiology (**Figure 1C**).

**Figure 2:**
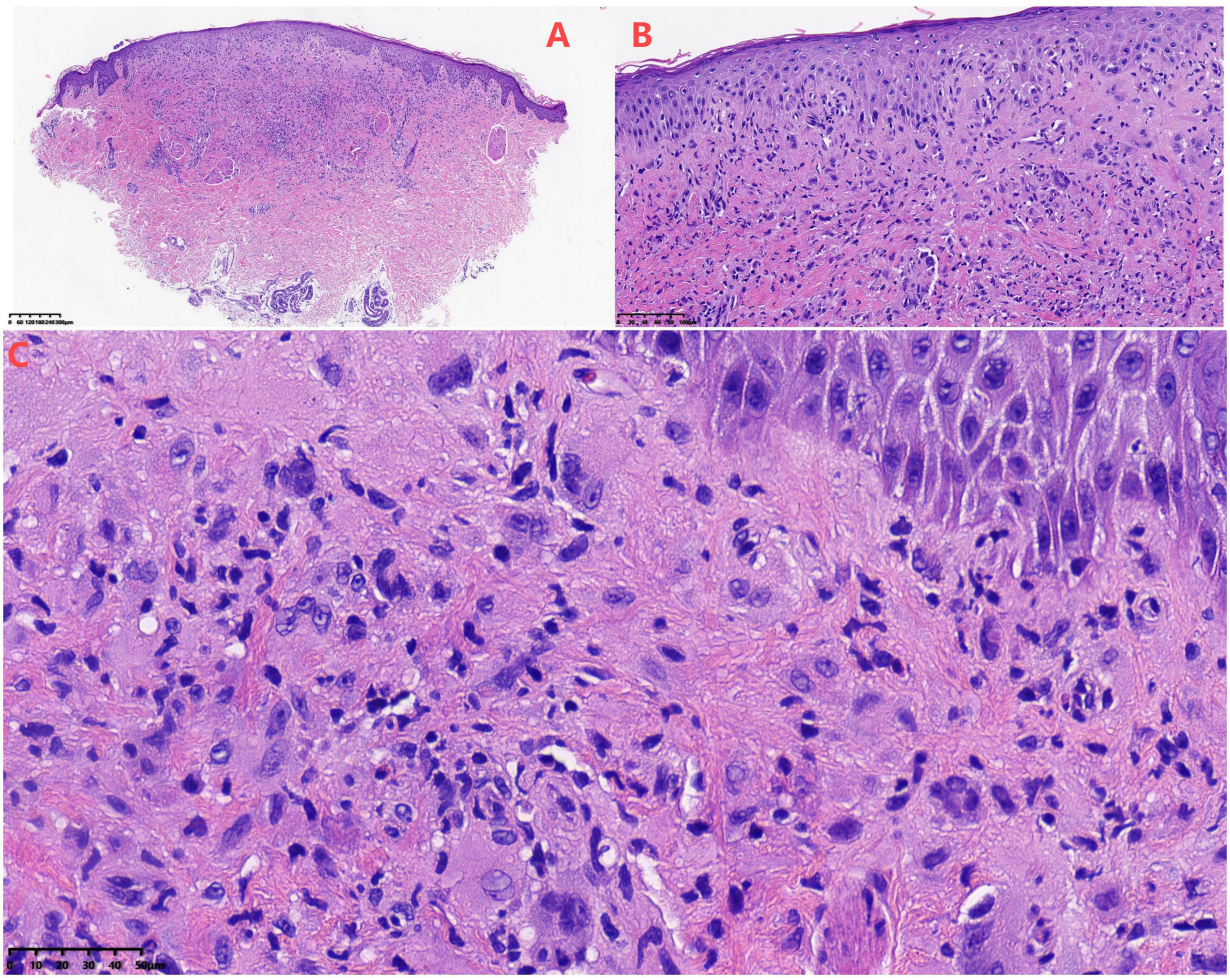
**(A)** Low-power view (hematoxylin and eosin staining, × 40) showing diffuse dermal infiltration composed of proliferating histiocyte-like cells, foam cells, and short spindle-shaped cells, with scattered multinucleated giant cells and lymphocytes. **(B)** Medium-power view (hematoxylin and eosin staining, × 200) demonstrating ill-defined histiocyte-like cells and foam cells; short spindle-shaped cells arranged in a scattered or fascicular pattern; and interspersed multinucleated giant cells and lymphocytes. **(C)** High-power view (hematoxylin and eosin staining, × 400) showing scattered histiocyte-like cells within the dermis, which feature prominent nucleoli and abundant eosinophilic cytoplasm, accompanied by interspersed multinucleated giant cells.

**Figure 3:**
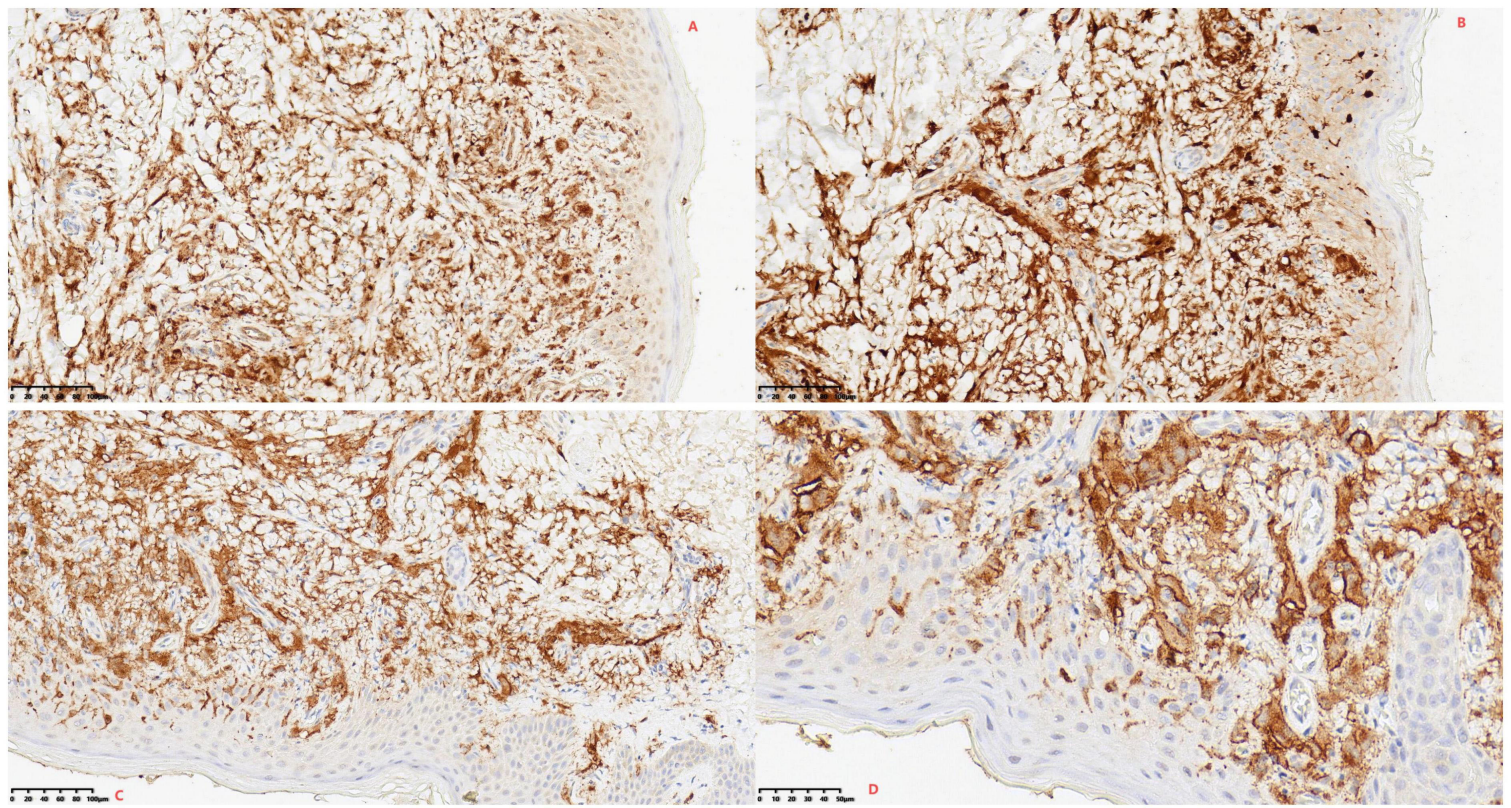
**(A)** Immunohistochemical staining reveals positivity for CD68 (hematoxylin counterstain, × 200). **(B)** Immunohistochemical staining reveals positivity for S-100 (hematoxylin counterstain, × 200). **(C)** Immunohistochemical staining reveals positivity for ALK (hematoxylin counterstain, × 200). **(D)** High-power view (ALK immunohistochemistry staining, × 400) showing cytoplasmic immunoreactivity, or concomitant nuclear and cytoplasmic immunoreactivity, in tumor cells. Immunohistochemical staining was performed using a ready-to-use mouse monoclonal antibody against ALK p80 (clone 5A4, catalog number: MAB-0281; Fuzhou Maixin Biotechnology Development Co., Ltd., Fuzhou, Fujian, China).

**Figure 4:**
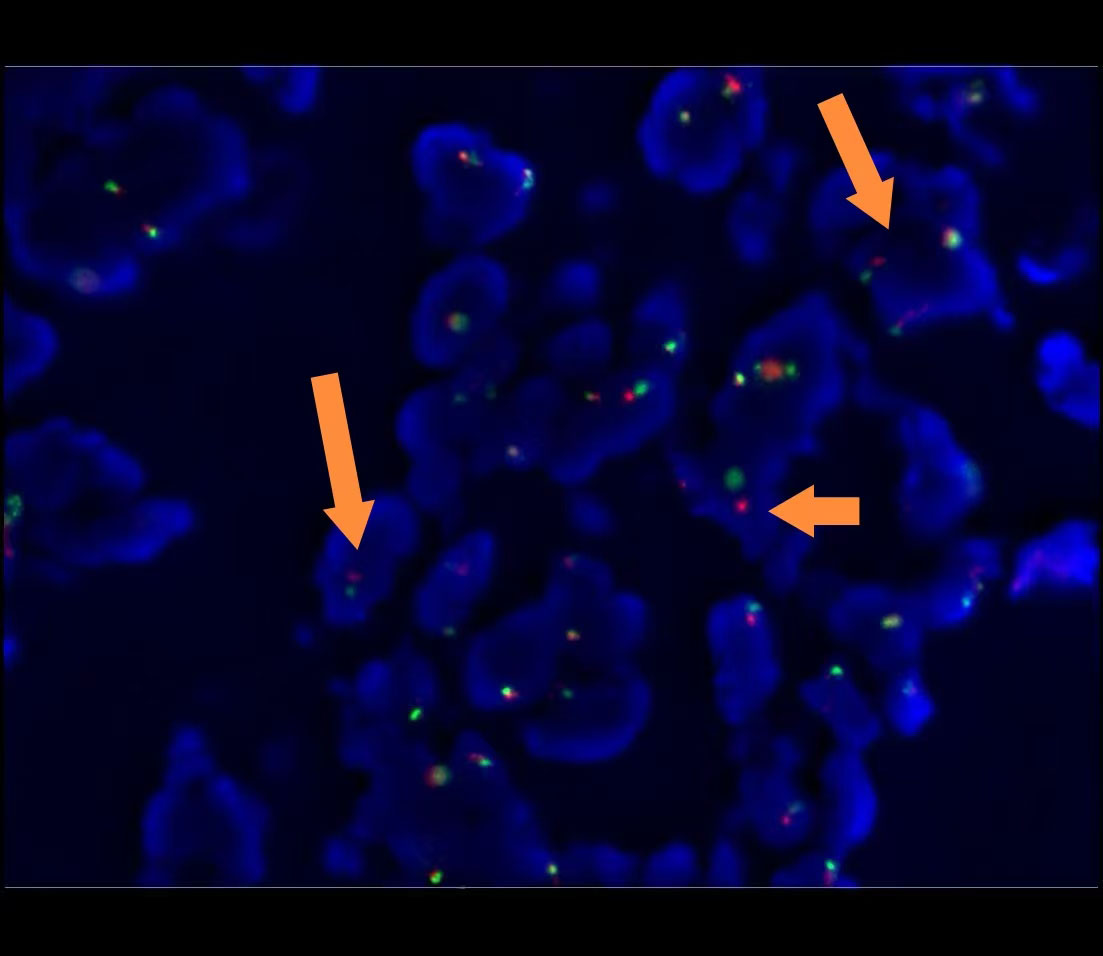
Break-apart FISH analysis demonstrates *ALK* rearrangement, indicated by one red, one green, and one yellow signal, or by separated red and green signals (The arrow indicates the location of the separated signals of the *ALK* gene).

Following failure of antimicrobial therapy, a biopsy of the lower extremity rash was performed. Histopathological examination revealed dermal proliferation of histiocytes, foamy cells, and short spindle cells, accompanied by scattered multinucleated giant cells and lymphocytes (**Figures 2A–C**). Immunohistochemical staining demonstrated positivity for CD68 (**Figure 3A**, magnification × 200), S100 (**Figure 3B**, magnification × 200), and ALK (**Figure 3C**, magnification ×200; **Figure 3D**, magnification × 400), and negativity for CD1a, CD3, CD20, CD30, myeloperoxidase (MPO), BRAF V600E, and Langerin. Break-apart FISH analysis confirmed *ALK* rearrangement (**Figure 4**).

NGS further identified a novel *PTRH2::ALK* fusion gene (**Supplementary Figure 1**). Whole-body positron emission tomography/computed tomography (PET/CT) revealed several key findings, including bilateral anterosuperior mediastinal thymic soft tissue masses (left: 13.5 × 6.1 × 7.1 mm, SUVmax = 3.7; right: 7.5 × 5.4 × 4.8 mm, SUVmax = 1.9), as well as a soft tissue lesion within the right T12/L1 intervertebral foramen (7.5 × 8.7 × 10.6 mm, SUVmax = 1.7). An irregular mass was identified at the porta hepatis (29.4 × 21.5 × 44.2 mm, SUVmax = 4.7), accompanied by multiple hypodense hepatic lesions, predominantly involving the right anterior lobe (SUVmax = 8.4). Additional findings included a hypermetabolic focus at the inferior pole of the right kidney (SUVmax = 4.1), a mesenteric nodule (SUVmax = 2.5), retroperitoneal lymphadenopathy with prominence at the right renal hilum (SUVmax = 3.2), and heterogeneous bone density, most notably in the left proximal tibia (SUVmax = 5.3).

Following initiation of treatment with the ALK inhibitor crizotinib, the patient maintained sustained clinical stability throughout the entire follow-up period. During follow-up, the pediatric patient remained afebrile, and the cutaneous lesions resolved completely. Inflammatory markers, including CRP, normalized to 2.93 mg/L. In addition, hemoglobin levels increased to within the normal range (128 g/L). Follow-up imaging using abdominal color Doppler ultrasonography demonstrated no evidence of hepatosplenomegaly and complete resolution of hypoechoic hepatic lesions.

## 3 Discussion

A literature search was conducted in the PubMed database using the keyword “ALK-positive histiocytosis” yielding 85 articles as of 2025. After systematic review and synthesis, 70 pediatric cases with detailed clinical data were identified and are summarized in **Table 1** (3, 5–20). The age of these patients ranged from the neonatal period to 17 years, including 40 females and 30 males. Based on lesion distribution, cases were classified as localized (n = 36) or systemic (n = 34). Genetically, reported *ALK* fusion genes included *CLTC::ALK*, *COL1A2::ALK*, *DCTN1::ALK*, *EML4::ALK*, *KIF5B::ALK*, and *TPM3::ALK*, among which *KIF5B::ALK* was the most frequently identified, accounting for 38 cases. Treatment strategies varied and included surgical resection, chemotherapy, hormone therapy, ALK inhibitors, or observation without intervention. Prognostic outcomes differed substantially: 49 patients experienced no recurrence, 5 patients died, 6 patients were alive with regressive disease, and the remaining cases lacked clearly documented prognostic information.

**Table 1:** Clinical characteristics of 70 pediatric cases with ALK-positive histiocytosis.

ID (reference)	Gender	Age at presentation	Disease extent (organs involved) (CNS: central nervous system; PNS: peripheral nervous system.)	Gene fusion	Treatment	Follow-up (NS, not sure; NR, no recurrence)
1 (7)	Male	3 years	Localized (external auditory canal)	Unknown	Resection	NR
2 (8)	Female	7 years	Localized (CNS)	*KIF5B-ALK*	Resection	NR
3 (8)	Female	10 years	Localized (CNS)	*KIF5B-ALK*	Resection	NR
4 (9)	Female	Neonate	Systemic (spleen, liver, bone marrow, skin)	*TPM3-ALK*	Chemotherapy (dexamethasone and etoposide)	NR (12 years)
5 (9)	Female	3 months	Systemic (spleen, liver, bone marrow)	Unknown	Chemotherapy (dexamethasone and etoposide)	NR (14 years)
6 (9)	Female	3 months	Systemic (spleen, liver, bone marrow)	Unknown	Antibiotics	NR (13 years)
7 (9)	Female	2 months	Systemic (spleen, liver, bone marrow)	*KIF5B-ALK*	Chemotherapy (dexamethasone and etoposide)	NR (2 years)
8 (9)	Male	3 months	Systemic (skin, kidney, lung, liver, bone marrow)	*KIF5B-ALK*	Chemotherapy (ALCL99 protocol)	NR (4 years)
9 (9)	Male	2 years 9 months	Systemic (intestine, bone marrow, central nervous system)	Unknown	Steroids and chemotherapy (etoposide, cyclosporine, immunoglobulins, cytarabin, methotrexate)	Died (2 months)
10 (9)	Male	2 years 3 months	Localized (nasal skin papule)	*KIF5B-ALK*	Biopsy followed by incomplete excision 6 months later	NR (2.5 years)
11 (9)	Male	15 years	Localized (cavernous sinus)	*KIF5B-ALK*	ALK-inhibitor (crizotinib)	NR (6 months)
12 (9)	Male	16 years	Localized (skin/soft tissue of foot)	*COL1A2-ALK*	Surgical resection (resection margin status uncertain)	NR (3 years)
13 (10)	Male	1 year 4 months	Systemic (CNS,lung)	*KIF5B-ALK*	Chemotherapy (cytarabine, vincristine, prednisolone, crizotinib)	Died (2 months)
14 (11)	Female	Neonate	Systemic (spleen,liver,bone marrow)	Unknown	Steroids and immunoglobulin	NR (6 months)
15 (12)	Male	3 years	Systemic (nervous system, paranasal mass, lungs, pancreas, spleen, appendix, lymph nodes, bones)	*KIF5B-ALK*	Unknown	NS
16 (13)	Male	1 month	Systemic (skin, liver, spleen, bone marrow)	Unknown	No intervention	NR
17 (14)	Male	1 month	Systemic (liver,bone marrow)	Unknown	Unknown	NS
18 (15)	Female	11 years	Localized (CNS)	*KIF5B-ALK*	Resection	NS
19 (16)	Female	4 years	Localized (nasal cavity)	Unknown	ALK-inhibitor	NS
20 (17)	Male	6 month	Localized (skin/soft tissue of left forearm)	*DCTN1-ALK*	Resection	NR
21 (18)	Male	4 month	Systemic (skin/soft tissue on back, trunk,hips, CNS, liver, parotid galands, kidneys, pelvis, bone)	Unknown	No intervention	NS
22 (6)	Male	2 years	Localized (Behind the right ear)	*KIF5B-ALK*	Resection	NR (15 months)
23 (6)	Female	2 years	Localized (prethorax)	*KIF5B-ALK*	Resection	NR (19 months)
24 (6)	Female	6 years	Localized (back)	*KIF5B-ALK*	Resection	NS
25 (6)	Male	3 months	Localized (prethorax)	Unknown	Resection	NR (14 months)
26 (6)	Female	8 years	Localized (Right corner of mouth)	Unknown	Resection	NR (3 years)
27 (6)	Male	1.5 years	Localized (torso)	Unknown	Resection, Chinese medicine	NR (9 months)
28 (6)	Female	6.5 years	Localized (torso)	*EML4-ALK*	ALK-inhibitor (Aletinib)	NR (6 months)
29 (6)	Female	3 months	Systemic (Skin, liver, lungs, vagina, lymph nodes, bone, brachial plexus, CNS)	*KIF5B-ALK*	ALK-inhibitor (Aletinib)	NR (2.5 years)
30 (6)	Male	3 years	Systemic (Submeningeal/multiple)	*KIF5B-ALK*	ALK-inhibitor (Aletinib)	NR (2.7 years)
31 (6)	Female	4 month	Systemic (Liver, lymph nodes, lungs, pancreas)	*KIF5B-ALK*	ALK-inhibitor (Aletinib)	NR (3 years)
32 (6)	Female	3 months	Systemic (Soft tissue, liver, spleen, pancreas, lung, kidney)	Unknown	ALK-inhibitor (Aletinib)	NR (2.5 years)
33 (6)	Male	6 years	Systemic (liver, bone marrow, testis)	*KIF5B-ALK*	Resection	Died
34 (19)	Female	2 months	Systemic (liver, bone marrow)	*CLTC-ALK*	ALK-inhibitor (Aletinib)	NR (1 month)
35 (5)	Female	Neonate	Systemic (liver, hematopoietic system, spleen, lung, possibly kidney)	Unknown	Steroids and immunoglobulin	NR (3.5 years)
36 (5)	Female	27 days	Systemic (liver, hematopoietic system, spleen,+ kidney at relapse)	Unknown	Chemotherapy (VBL/PRED)	NR (7 years)
37 (5)	Male	1 month	Systemic (liver, hematopoietic system, spleen, kidney, skin)	*KIF5B-ALK*	Chemotherapy (VBL/DEX/MTX)	Died (1 month)
38 (5)	Female	2 months	Systemic (liver, hematopoietic system, spleen)	Unknown	Supportive care	NR (4.5 years)
39 (5)	Female	4 months	Systemic (liver, hematopoietic system)	Unknown	Unknown	NS
40 (5)	Male	5 months	Systemic (liver, hematopoietic system, spleen)	*CLTC-ALK*	Chemotherapy (PRED/VBL)	NR (4 years)
41 (5)	Female	3 months	Systemic (Bone, lung, liver)	*TPM3-ALK*	Chemotherapy (PRED/VBL/6MP)	NR (2 years)
42 (5)	Female	9 months	Systemic (lung, skin, kidney)	Unknown	Chemotherapy (PRED/VBL)	NR (2 years)
43 (5)	Male	10 months	Systemic (CNS, lung, liver, soft tissue (peritoneum))	*KIF5B-ALK*	Chemotherapy (PRED/VBL), combined with ALK inhibition (alectinib)	NR (13 months)
44 (5)	Female	2 years	Systemic (CNS, bone, lung, liver, skin, soft tissue (perineal mass), lymph node, kidney, breast, pancreas)	*KIF5B-ALK*	Chemotherapy (PRED/VBL) with ALK inhibition (lorlatinib)	Alive with regressive disease (21 months)
45 (5)	Female	10 years	Systemic (CNS, lung, lymph node, cervix, thyroid, submandibular salivary gland)	*KIF5B-ALK*	ALK-inhibitor (crizotinib)	NR (2 years)
46 (5)	Female	7 months	Localized (CNS)	*KIF5B-ALK*	Corticosteroids, followed by ALK inhibition (lorlatinib)	Alive with regressive disease (5 months)
47 (5)	Female	9 months	Localized (CNS)	Unknown	Unknown	unknown
48 (5)	Male	2.5 years	Localized (CNS)	*KIF5B-ALK*	Chemotherapy (Headstart protocol, 1 cycle), followed by ALK inhibition (alectinib)	NR (16 months)
49 (5)	Female	3 years	Localized (CNS)	*KIF5B-ALK*	No intervention	NR (12 months)
50 (5)	Female	3 years	Localized (PNS)	*KIF5B-ALK*	Corticosteroids, followed by surgery and chemotherapy (VBL/PRED)	Alive with regressive disease (2.5 years)
51 (5)	Female	7 years	Localized (CNS)	*KIF5B-ALK*	Chemotherapy (Clofarabine)	NR (6 months)
52 (5)	Female	11 years	Localized (CNS)	*KIF5B-ALK*	Resection	NR (9 months)
53 (5)	Male	11 years	Localized (PNS)	Unknown	Resection	NR (18 years)
54 (5)	Male	12 years	Localized (PNS)	*KIF5B-ALK*	Resection, followed by chemotherapy (VBL/PRED)	NR (2.5 years)
55 (5)	Female	13 years	Localized (CNS/PNS)	*KIF5B-ALK*	Resection, followed by ALK inhibition (alectinib)	Alive with minor, regressive disease (2.5 years)
56 (5)	Female	6 months	Localized (skin)	*KIF5B-ALK*	Resection	NR (2 years)
57 (5)	Male	7 months	Localized (skin)	*KIF5B-ALK*	Resection	NR (2 years)
58 (5)	Female	21 months	Localized (skin)	*KIF5B-ALK*	Resection	NR (2 years)
59 (5)	Male	2 years	Localized (soft tissue)	*KIF5B-ALK*	No intervention	Alive with regressive disease (14 months)
60 (5)	Female	3 years	Localized (soft tissue)	*KIF5B-ALK*	Resection	NR (14 months)
61 (5)	Male	3 years	Localized (soft tissue)	*KIF5B-ALK*	Corticosteroids, followed by resection	NR (3 years)
62 (5)	Female	10 years	Localized (skin)	*KIF5B-ALK*	Resection	NR (1 months)
63 (5)	Female	10 years	Localized (Bone)	*KIF5B-ALK*	Resection	NR (5 years)
64 (5)	Male	11 years	Localized (soft tissue)	*KIF5B-ALK*	Resection, followed by chemotherapy (VBL/PRED)	Alive with stable disease (15 months)
65 (5)	Male	17 years	Localized (lung)	*EML4-ALK*	Unknown	unknown
66 (20)	Male	18 months	Systemic (CNS,lung)	*KIF5B-ALK*	Resection, followed by chemotherapy (CHOP) and ALK inhibition (crizotinib)	Died (8 months)
67 (14)	Female	Neonate	Systemic (liver, kidney, bone marrow)	Unknown	Unknown	NS
68 (3)	Female	Neonate	Systemic(liver, hematopoietic system)	*TPM3-ALK*	Corticosteroids, and chemotherapy (etoposide)	NR (2.5 years)
69 (3)	Female	3 months	Systemic(liver, hematopoietic system)	Unknown	Corticosteroids, and chemotherapy (etoposide)	NR (5 years)
70 (3)	Female	3 months	Systemic(liver, hematopoietic system)	Unknown	No intervention	NR (7 years)

In this article, we report the case of an 8-month-old male infant diagnosed with the systemic subtype of ALK-positive histiocytosis, with involvement of the skin, liver, hematopoietic system, mediastinum, right kidney, mesentery, bones, and lymph nodes. Notably, the diagnostic process was prolonged and challenging. Initial abdominal ultrasonography and CT revealed a hepatic mass suspicious for a liver abscess, and blood tests demonstrated elevated inflammatory markers suggestive of infection. These findings were highly misleading and resulted in an initial misdiagnosis of liver abscess. Following ineffective anti-infective therapy, the definitive diagnosis was ultimately established through pathological examination of a skin lesion.

In this patient, the rash was localized to the bilateral lower extremities, including the plantar surfaces. According to the family’s report, the rash had been present for more than 2 months, characterized by spontaneous regression with a tendency to recur. Clinically, the rash consisted of multiple discrete papules and macules that were pale red or skin-colored, with relatively regular morphology and a scattered distribution. A review of previously reported cases with cutaneous involvement indicates that skin manifestations of ALK-positive histiocytosis are nonspecific (5, 6, 8, 13, 17, 18). This lack of specificity poses a challenge to clinical diagnosis and underscores the critical importance of pathological evaluation.

The pathological diagnostic criteria for ALK-positive histiocytosis require histologic confirmation of histiocytosis, with lesional cells that expressing macrophage/histiocyte markers, including CD163, CD68, CD14, and/or CD4 (5). In the present case, the immunophenotypic profile was consistent with these criteria, and ALK expression was confirmed. Furthermore, molecular testing demonstrated *ALK* gene rearrangement. Notably, a previously unreported *ALK* fusion gene, *PTRH2::ALK*, was identified.

In humans, the *PTRH2* gene is located on chromosome 17 and is ubiquitously expressed across tissues. In oncologic contexts, *PTRH2* has been reported to function as a putative oncogene that promotes malignant progression and metastatic dissemination. In addition to regulating Bcl-2 expression, *PTRH2* modulates the phosphatidylinositol 3-kinase/protein kinase B (PI3K/AKT) and extracellular signal-regulated kinase (ERK) signaling pathways, thereby influencing key cellular processes involved in adhesion-dependent signaling, including cell survival, proliferation, and differentiation (21). The *PTRH2::ALK* fusion has previously been identified in patients with inflammatory myofibroblastic tumor (IMT), but has not been reported in ALK-positive histiocytosis (22). Beyond expanding the molecular spectrum of ALK-positive histiocytosis, the identification of this novel ALK fusion further supports the therapeutic relevance of ALK-targeted treatment strategies, particularly the use of ALK inhibitors, within the disease management framework (1). Among the 70 previously reported pediatric cases of ALK-positive histiocytosis, 16 patients received ALK inhibitors therapy. Of these, four were treated with crizotinib, nine with alectinib, and two with lorlatinib (5, 6, 9, 10, 19, 20). The specific ALK inhibitor administered to one additional patient was not specified (16).

Given the young age of the patient and the presence of multisystem involvement, treatment with the ALK inhibitor crizotinib was initiated. During the follow-up period, the patient’s clinical symptoms resolved, and laboratory parameters demonstrated marked improvement.

## 4 Conclusion

In conclusion, we report a case of ALK-positive histiocytosis harboring a novel *PTRH2::ALK* fusion that initially masqueraded as a liver abscess in an infant. The diagnostic challenges encountered highlight the necessity and complexity of pathological assessment in patients presenting with cutaneous manifestations. Given the rarity of ALK-positive histiocytosis, comprehensive diagnostic evaluation is essential for both exclusion and confirmation of this disease in patients with an unidentified hepatic mass.

## Data Availability

The original contributions presented in the study are included in the article/**Supplementary Material**. Further inquiries can be directed to the corresponding authors.

## Supplementary material

Supplementary Figure 1 Schematic representation of the *PTRH2::ALK* fusion identified by NGS.
