# Supplementary figures and images for: Case Report: ALK-positive histiocytosis with a novel PTRH2::ALK fusion masquerading as a liver abscess in an infant
Source: Front Immunol. 2026 Apr 29;17:1722061. doi: 10.3389/fimmu.2026.1722061 (PMC13167517; doi:10.3389/fimmu.2026.1722061)

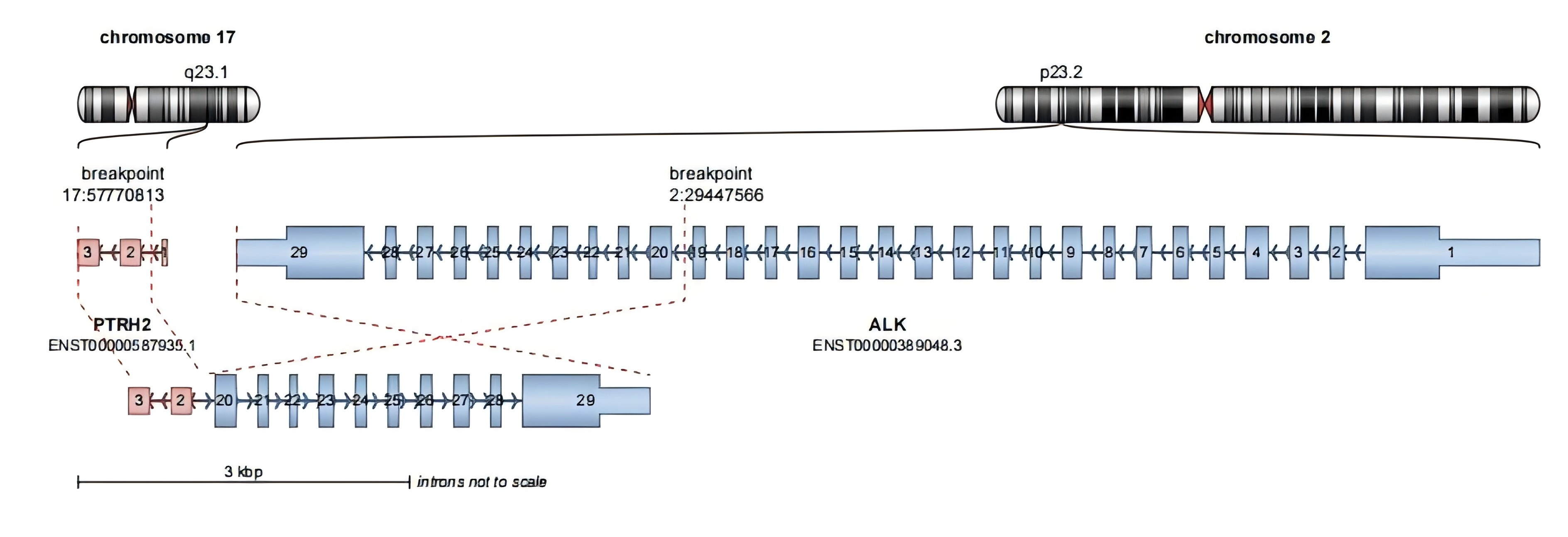

Supplement: Supplementary Figure 1 — Schematic representation of the PTRH2::ALK fusion identified by NGS. [file Image1.jpeg]
